# Texas Promoting Social Hygiene

**Published:** 1914-07

**Authors:** 


### Department of Eugenics.

Conducted by Dr. Malone Duggan and Dr. Theo. Y. Hull.

### THE TEXAS STATE SOCIETY OF SOCIAL HYGIENE.

Headquarters: San Antonio, Texas.

DR. MALONE DUGGAN, President, San Antonio.
PROF. A. CASWELL ELLIS, 2d Vice President, Austin.
MRS. ELI HERTZBERG, 3d Vice President, San Antonio.
DR. THEO. Y. HULL, Secretary, San Antonio.
MR. W. L. EVANS, Treasurer, San Antonio.

EXECUTIVE COMMITTEE.

Dr. Malone Duggan, Chm.
Dr. Theo. Y. Hull, Sec'y.
Dr. W. F. McCaleb.
Hon. C. H. Jenkins.
Rev. A. W. S. Garden.
Hon. J. C. Willacy.
Mr. W. L. Evans.
Dr. Frank D. Boyd.
Dr. J. R. Nichols.
Dr. J. M. McCutchan.
Dr. W. A. King.
Dr. J. W. Torbett.
Dr. F. C. Walsh.
Dr. Joe Reuss.
Dr. Frank Paschal.

### Texas Promoting Social Hygiene.

Mayor A. P. Wooldridge, of Austin, called a meeting to be held in his city June 9th in the interest of promoting social hygiene in this State. A formal organization will be effected in the fall in time to get ready for the next Legislature. This movement is in line with the American Society of Social Hygiene, and it deserves the support of all interested parties. The Texas State Society of Social Hygiene under the auspices of the State Medical Association has done pioneer work along educational lines, but owing to the apathy of the profession it has not been very active in the past year. It was instrumental, however, in interesting the Mothers' Clubs of the State, which have carried on the work in a much more effective manner.

